# The Origins and Risk Factors for Serotype-2 Vaccine-Derived Poliovirus Emergences in Africa During 2016–2019

**DOI:** 10.1093/infdis/jiad004

**Published:** 2023-01-11

**Authors:** Elizabeth J Gray, Laura V Cooper, Ananda S Bandyopadhyay, Isobel M Blake, Nicholas C Grassly

**Affiliations:** Department of Infectious Disease Epidemiology, Imperial College London, London, United Kingdom; Department of Infectious Disease Epidemiology, Imperial College London, London, United Kingdom; Bill and Melinda Gates Foundation, Seattle, Washington, USA; Department of Infectious Disease Epidemiology, Imperial College London, London, United Kingdom; Department of Infectious Disease Epidemiology, Imperial College London, London, United Kingdom

**Keywords:** polio, cVDPV2s, emergence risk, mOPV2, mOPV2 campaign risk, nOPV2 prioritization

## Abstract

Serotype 2 oral poliovirus vaccine (OPV2) can revert to regain wild-type neurovirulence and spread to cause emergences of vaccine-derived poliovirus (VDPV2). After its global withdrawal from routine immunization in 2016, outbreak response use has created a cycle of VDPV2 emergences that threaten eradication. We implemented a hierarchical model based on VP1 region genetic divergence, time, and location to attribute emergences to campaigns and identify risk factors. We found that a 10 percentage point increase in population immunity in children younger than 5 years at the campaign time and location corresponds to a 18.0% decrease (95% credible interval [CrI], 6.3%–28%) in per-campaign relative risk, and that campaign size is associated with emergence risk (relative risk scaling with population size to a power of 0.80; 95% CrI, .50–1.10). Our results imply how Sabin OPV2 can be used alongside the genetically stable but supply-limited novel OPV2 (listed for emergency use in November 2020) to minimize emergence risk.

## Teaser

Outbreaks of vaccine-derived poliovirus from rare reversion of type-2 Sabin vaccine strain inhibit polio eradication efforts. We use a hierarchical model, incorporating VP1 region genetic divergence and population movement, to estimate outbreak origins and risk factors for vaccine campaigns.

In April 2016, the routine immunization of children with tOPV (trivalent Sabin oral polio vaccine, against poliovirus serotypes 1, 2, and 3) was ended worldwide, replaced by bOPV (bivalent oral polio vaccine, against serotypes 1 and 3) to reduce the risks of emergence of new serotype-2 vaccine derived polioviruses (VDPV2s) [1]. In mainland Africa, since then there have been environmental detections and/or cases of VDPV2s from 56 distinct emergence groups, 51 of which are estimated to have emerged after April 2016. Paradoxically, the vaccination campaigns from which these emergence groups are likely to have emerged were carried out over a limited area as targeted responses to previous emergences. The breaking of this case-response-emergence cycle is essential for eradication of circulating VDPV2s (cVDPV2s). Despite emergency use listing of a genetically-stable novel OPV (nOPV2) in November 2020 [2], limited supply relative to demand could necessitate continued Sabin OPV use in certain instances. For eradication to be achieved, nOPV2 must be prioritized for regions with the highest risk of VDPV2 emergence and all responses must be conducted as to minimize the risk of a new emergence.

The probability of an emergence depends on many factors. Incidence of cVDPV2 detection is higher where there is lower mucosal immunity [3]. Prior to the tOPV withdrawal, it was predicted via simulation and analytical models that given a low baseline of immunity, a small number of campaigns may actually increase the probability of a cVDPV2 emergence [4]. Mathematical model-based simulation of vaccination campaigns (supplementary immunization activities, SIAs) carried out in provinces of Nigeria [5] also predicted that the probability of a new emergence would depend on preexisting levels of mucosal immunity, predicting a need for large-scale aggressive vaccination responses to any new emergences in the 18 months after tOPV withdrawal. The frequency of viral shedding after receiving an OPV dose is inversely proportional to the preexisting level of mucosal immunity [6]. Thus, low levels of preexisting OPV-induced serotype-2 immunity in a population are correlated with persistent detection of Sabin viruses following an SIA [7], giving more opportunity for reversion to a VDPV2.

Lack of access to good sanitation may also contribute, facilitating sustained fecal-oral transmission of vaccine strains and cVDPV2s [6]. Locational connectivity, trade, displacement, etc. will affect the spatial scale of transmission and emergence risk [8, 9]. Analytical modeling has shown that campaign size is important, identifying a peak in emergence risk for intermediate campaign size: small campaigns are of low risk because of the small number of individuals given OPV, as are very large campaigns, because of lower probability of sustained transmission with fewer susceptible individuals [4]. In this article we seek to better understand the process of emergence and its risk factors by estimating which campaigns were likely to have caused cVDPV2 emergences in Africa using a Bayesian hierarchical model fit to data on the timing, location, and genetic divergence of the earliest cases or detections of all observed emergence groups. We use a radiation model to capture population movement and assume viral evolution in the VP1 region of the poliovirus genome is a Poisson process. This allows us to examine possible connections between risk factors and whether a campaign was likely to have led to an emergence. The attribution of cVDPV2 emergences to campaigns also highlights gaps in surveillance, where an emergence has been first detected far, spatially or temporally, from its source. The closure of these gaps is essential for timely emergence response and the limitation of spread [8, 10].

## METHODS

### Data

#### Virus Detections

We take the set of cVDPV2 detections (poliomyelitis cases, environmental detections, healthy contact survey detections) from emergence groups detected between 1 April 2016 and 31 December 2021. A VDPV2 is defined as an OPV virus strain with >0.6% genetic divergence in the VP1 genomic region, while a cVDPV2 must have evidence of person-to-person transmission [11]. An emergence group is defined as all genetically related isolates of cVDPV2 (from children with acute flaccid paralysis [AFP], healthy children, or the environment). Emergence groups are defined by the Global Polio Eradication Initiative based on phylogenetic analysis to estimate whether groups of vaccine-derived viruses share a common origin, and are reported in the Polio Information System (POLIS), maintained by the World Health Organization (WHO) [12]. Restricting to emergences that, according to the number of nucleotide changes from the type-2 Sabin strain of the earliest 3 detections, have a greater than 50% chance of having emerged from a campaign carried out between April 2016 and December 2019 leaves 46 groups. The 2-year gap (which we would expect to correspond to approximately 21 nucleotide changes) since the end of this window gives reasonable confidence in our having observed at least 1 case or detection relating to every emergence from serotype-2 OPV used in this time window (most new cVDPV2 emergences have been detected within 2 years of their likely emergence based on their genetic divergence from Sabin poliovirus) [13].

#### Vaccination Campaign Data

SIAs carried out in mainland African countries between 1 April 2016 and 1 December 2019 were considered. We assume that all emergence groups first detected in mainland Africa were triggered by OPV used there. We subdivide each SIA by province, considering each province-SIA separately. The set of province and district boundaries current as of December 2021 were used. Changes in administrative boundaries occasionally mean these differ from boundaries used at the times of campaigns. To have a single, consistent set of campaign locations over time (necessary for modeling purposes) in these cases we assumed the campaign was carried out in the current province which contains the population-weighted centroid of the administrative district that applied at the time of the campaign.

#### Risk Factors

We considered whether the province-level proportion of children younger than 5 years with type-2 OPV-induced immunity during the 6-month period in which the campaign was conducted was associated with VDPV2 emergence. Immunity estimates were based on reported OPV doses by nonpolio AFP cases [14]. Campaign history data (timing, geography, and vaccine formulation) were used to scale reported vaccination histories by the proportion of campaigns in the child's lifetime that contained type-2 OPV (trivalent or monovalent OPV2). Other risk factors considered were estimated province-level diarrhoea prevalence [14]; the proportion of districts within the province that passed lot quality assurance sampling (LQAS) standards for campaign coverage (<4/60 children unvaccinated, giving a <5% chance of <90% coverage [15]); and the log of the whole population size of the area targeted by the whole campaign (not province campaign). Where LQAS data were unavailable (78% of province-campaigns) we used the mean proportion of districts within a province achieving a “pass” rating, (70.4%). Covariates are denoted *x_u_*_5_, *x_dp_*, *x_LQAS_*, *x_size_*. We attempted to include IPV-induced immunity [14] as a risk factor; however, its post-switch inverse relationship with OPV immunity rendered this infeasible. Additionally, IPV primarily induces a humoral response unless there has been previous OPV exposure or poliovirus infection, after which IPV boosts mucosal immunity. During the analysis period few IPV campaigns were conducted [14] meaning most IPV immunity would have been humoral, arising from 1 routine immunization dose.

#### Data Sources

Data on campaigns, virus detections, and LQAS were taken from POLIS [12]. Population data were extracted from WorldPop's 2020 rasters ([16, 17]). Other covariates were found in Cooper et al [14].

### Model Description: Attributing Outbreaks to Campaigns and Estimating Risk Factors

We developed a hierarchical modeling framework to construct a probability distribution of causal campaigns leading to each outbreak and estimate risk factors for outbreaks. The modeling framework draws together knowledge about the time, location, and nucleotide changes of the detections from each emergence group. We account for the fact that the initial detections may arise far from the causal campaign and for temporal delay before detection by incorporating the likely length of time between transmissions and locations between which a chain of transmission could reasonably have travelled. We present an overview of our approach, with detailed modeling methods found in the Supplementary Material.

The model consists of 3 main components: risk factor-informed relative campaign risks and spatially and temporally informed likelihoods. The risk factor component consists of a relative risk associated with each province-campaign of having caused any particular outbreak. We define parameters *θ_u_*_5_, *θ_dp_*, *θ_LQAS_*, *θ_size_* associated with the covariates *x_u_*_5_, *x_dp_*, *x_LQAS_*, *x_size_*. For province campaign *j* the risk is assumed proportional to *wj* exp(*θ_size_ log(x_size_, j) + θ_u5_x_u5_, j + θ_LQAS_x_LQAS_, j +. . .*), where *wj* is the proportion of the total population size targeted by the whole campaign that reside in the specific province *j*. This ensures that the model is not sensitive to province divisions, while the parameter *θ*_size_ controls for how risk grows with size (1, linearly; <1, larger campaigns have a lower per person risk). The other coefficients *θ*_u5_, etc. control for the importance of their corresponding risk factors. We normalize over the set of all province-campaigns to find a probability that each caused a particular outbreak, based on risk factors and their coefficients (Eq 1, Supplementary Material).

The temporal component of the model consists of 2 parts: the first assigning a likelihood to each campaign for each outbreak, based on the number of nucleotide changes in the first 3 (where available) detections. Nucleotide changes in the VP1 region may be assumed to occur at a roughly constant rate of once every 35.5 days (ie, a Poisson process with rate *λ* = 0.0281) [13, 18]). This implies the likelihoods of each campaign to be proportional to *P_E_* (*T* | *λ*, *nt*), an Erlang distribution with rate λ, shape the number of nucleotide changes *nt*, and *T* the gap in days between the campaign and detection. *P_E_* (*T* | *λ*, *nt*) is assumed to be zero for campaigns after the detection.

The time between each campaign and detection is secondly used to calculate a distribution of the number of generations through which the chain of transmission would have gone in that time gap *T*, assuming generation time follows a gamma distribution with mean 10 days [19]. This distribution of the number of generations informs the spatial component of the model. We model spatial movement by assuming a constant (unknown) “province retention” probability *β* of any transmission being between individuals of the same province. Conversely, given an out-of-province transmission, the probability of movement into any specific province is proportional to the value of the radiation model of population mobility [20] corresponding to the “sending” and “receiving” provinces. These step-by-step transmissions form a Markov process; exploitation of the Markov process properties facilitates finding the likelihood of a chain of transmission having travelled between campaign and detection locations within the generations implied by the time gap between them.

Combining these risk factor-implied relative risks with spatial and temporal likelihoods yields a posterior distribution of causal campaigns for each outbreak. Fitting such a model via Markov Chain Monte Carlo (jointly for all outbreaks, using all to inform parameter estimates) gives estimates for *β*, *θ_u_*_5_, *θ_dp_*, *θ_LQAS_*, and *θ_size_*, along with these distributions for each outbreak. Modeling details are in the Supplementary Material.

Due to the computational intensity of such a model fitting procedure, it was not feasible to carry out a highly rigorous model selection process, or indeed to fit models on all subsets of possible risk factors. Nonetheless, to mitigate against unnecessary levels of uncertainty in parameters associated with covariates that are clear predictors of outbreak causation, we included only parameters with effects that were consistently and discernibly nonzero, specifically those with either <5% or >95% of their probability mass above zero. In a full, all-covariate, model the parameters associated with diarrhea prevalence and LQAS had 71% and 74% of their probability mass above zero, and were thus excluded.

## RESULTS

### mOPV2 Campaigns Causing cVDPV2 Emergences

**Figure 1.**
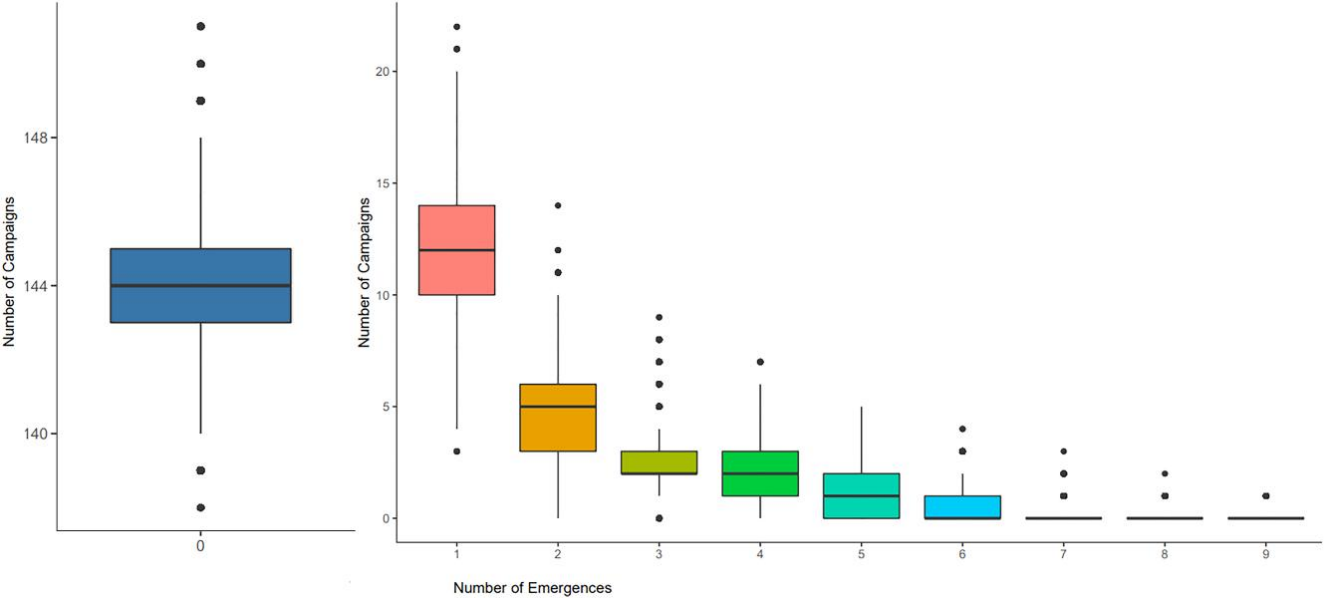
Posterior distributions of the number of emergences per campaign, from the 40 000 sets of model output campaign-assignments; for example, an average of 12 campaigns led to 1 emergence each. Boxplots show the median, upper and lower quartiles, range and outliers.

**Figure 2.**
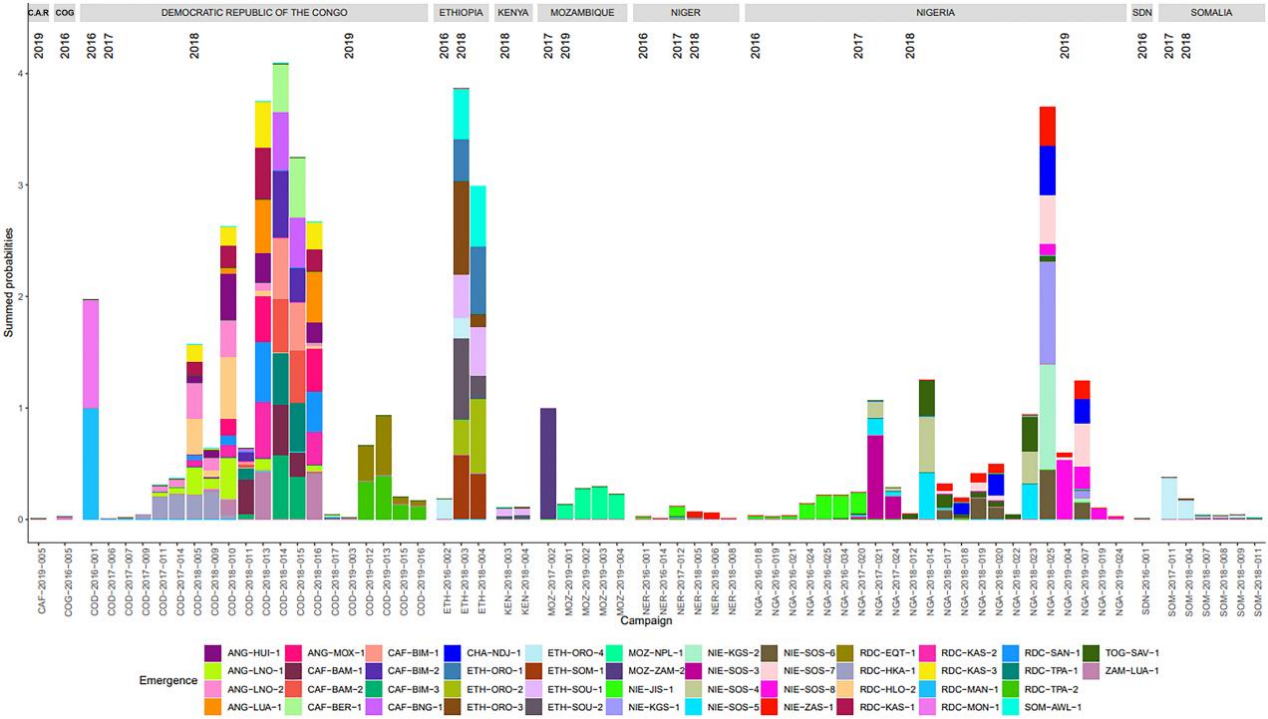
Attribution of emergences to likely campaigns: the height of the correspondingly colored section of a bar represents the probability of an emergence having been caused by the campaign on the x-axis. We show supplementary immunization activities for which the sum of probabilities is ≥ .01. Thus, the bar sections corresponding to a particular color sum to close to one. Each campaign has a unique code as indicated on the x-axis, based on the 3-letter code country, year, and campaign number. Emergence codes are based on place names. Abbreviations: ANG, Angola; CAF, Central African Republic; COG, Republic of the Congo; CHA, Chad; ETH, Ethiopia; MOZ, Mozambique; NER, Niger; NIE/NGA, Nigeria; RDC/NIE, Democratic Republic of the Congo; SDN, Sudan; SOM, Somalia; TOG, Togo; ZAM, Zambia.

**Figure 3.**
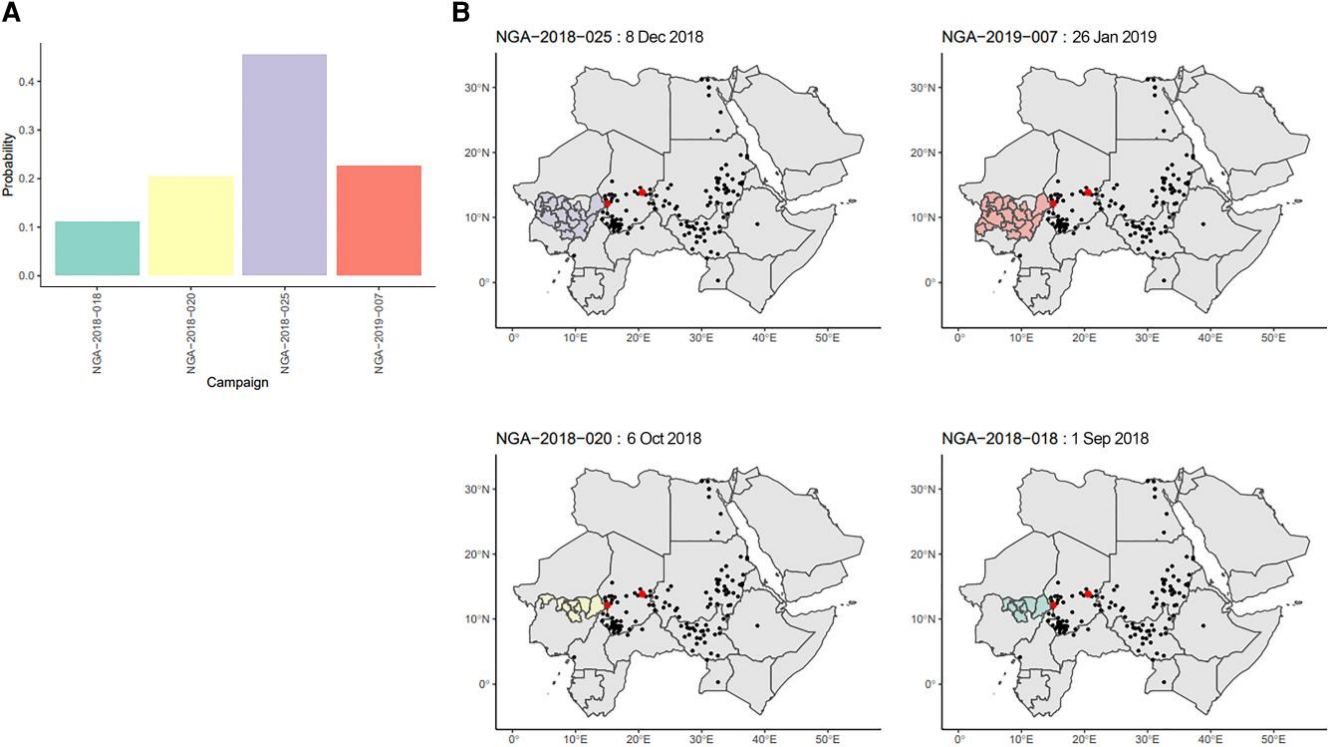
*A*, Posterior probabilities for campaigns with ≥.01 probability of causing the CHA-NDJ-1 emergence, detected in N’Djamena, Chad, in August 2019, with 7 nucleotide changes from Sabin. *B*, Maps of the location of the candidate campaigns and subsequent detections of the CHA-NDJ-1 lineage in people or the environment, demonstrating possible range of spread. Shaded areas indicate the areas targeted by campaigns. Red points indicate locations of districts of the first 3 detections. Black points indicate subsequent detections. Abbreviation: NGA, Nigeria.

Of the 167 campaigns considered, 150 were not estimated to be the most likely campaign for any emergence. The distribution of numbers of likely emergences per campaign is shown in Figure 1. Additionally, 100 campaigns had a sum of emergence-causation probabilities (ie, the probability that the campaign caused the emergence, summed over all emergences, which we use as an indicator of aggregate risk) of ≤.01. The causation probabilities for the remaining 67 campaigns are shown in Figure 2. We see that most SIAs are estimated as extremely unlikely to have caused any emergences, with large numbers of emergences having similar sets of likely causal campaigns. The distribution of likely campaigns for the CHA-NDJ-1 emergence is shown by way of illustration in Figure 3.

### Risk Factors

**Figure 4.**
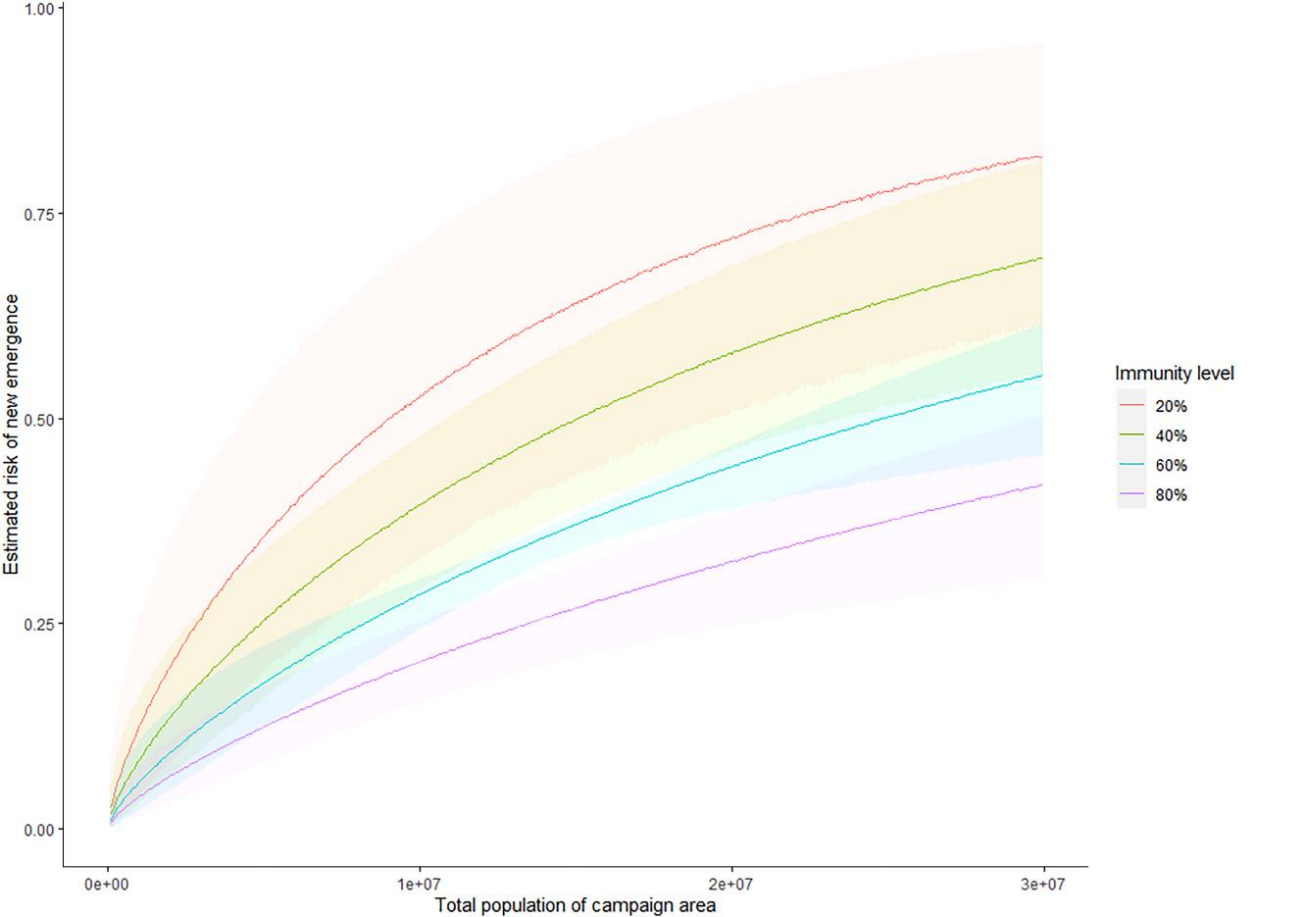
Estimated risks of new emergences, with 95% credible intervals, shown in the shaded areas, given population and immunity levels in children younger than 5 years. These are calculated as described in Supplementary Material Section 4.

Parameter estimates are displayed in Table 1: retained covariates were population immunity and campaign size. The estimate of *θ*_u5_ = −1.98 indicates increased risk in campaigns carried out in places of low immunity and may be interpreted such that given 2 otherwise identical campaigns, one in a location of 80% immunity and the other 20%, we would consider the one of 20% to be (exp[ − 1.98 × 0.2])/(exp[ − 1.98 × 0.8]) = 3.28 times more likely to be the causal campaign of an outbreak, or that a 10 percentage point increase in immunity is associated with a 1 − exp(0.1 × 1.98) = 18.0% decrease in relative risk of emergence from a campaign. Similarly, the estimate of *θ*_size_ = 0.795 indicates a larger campaign has higher risk than a smaller campaign, but a lower per-person risk. Given campaigns in locations of total populations 1 million and 10 million, we would consider the larger to be 10^0.795^= 6.23 times riskier than the smaller. Using these fitted values and the total number of observed emergences we derive an estimate for the per person-in-population risk, *U*, for a campaign across an area with population *p* and estimate of immunity in children younger than five years *q* of *U* = 3.10 *×* 10^−6^ exp(−0.205 log(*p*) − 1.98*q*). We infer that a campaign in an area with population 1 million with a 5% level of immunity amongst children younger than 5 years has an outbreak-causation probability *U* = 16.5% (95% credible interval [CrI], 4.73%–35.9%). For a similarly sized campaign with an immunity level of 50% this becomes *U* = 6.80% (95% CrI, 2.68%–12.9%). Estimated probabilities for different immunity levels and population sizes are shown in Figure 4.

**Table 1.** Posterior Estimates of Model Parameters Relating to Speed of Spread and the Impact on the Risk of Emergence That Size and Under 5 Type-2 Population Immunity Has

Parameter	Mean Value (95% CrI)
*β,* province retention probability	0.957 (.946 to .967)
*θ_u_*_5_, type-2 immunity in population in younger than 5 y	−1.98 (−3.31 to −.647)
*θ_size_,* campaign area population size	0.795 (.503 to 1.10)

The risk of a new emergence being from a campaign over a place with population *x_size_* and proportion of children younger than 5 years with immunity *x_u5_* is proportional to exp(0.795 log[*x_size_*] − 1.98*x_u5_*). The 95% credible interval (CrI) is the interval in which 95% of the probability mass of that parameter lies: viewed as a random variable, the parameter has a 95% chance of being found there.

### Time and Distance Between the Originating Campaign and Detection of a cVDPV2

**Figure 5.**
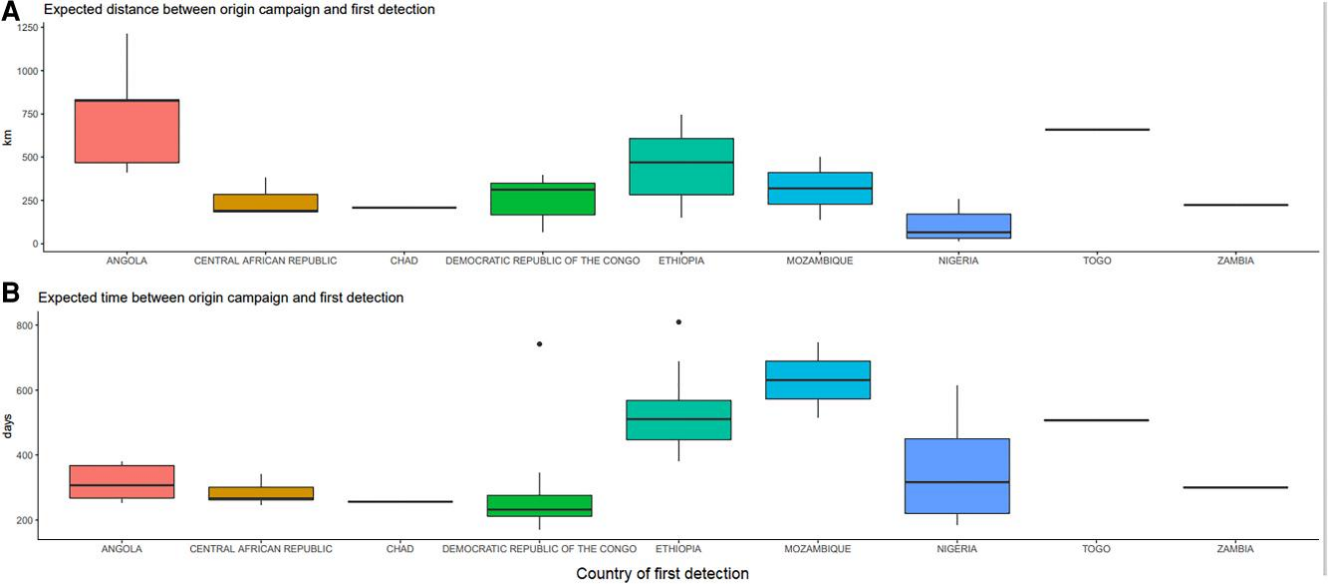
Expected distances (km) and times (days) between the causal campaign and the first detection, by country. Distances are between the centroid of the province of the province-supplementary immunization activities and the centroid of the district of first detection.

Using posterior samples of likely causal campaigns, we find the expected time and distance (details in Supplementary Material) between the campaign and the first detection of the emergence. This suggests most emergences are detected within 14 months (25%–75% quantiles of 246–453 days) and 450 km (25%–75% quantiles of 151–397 km) of the likely causal campaign (Figure 5). Notable exceptions are emergences in Ethiopia and Mozambique taking longer to be detected, and the 2019 Angola emergences detected an unusually long way from the campaigns to which they have been attributed.

## DISCUSSION

We find that a small number of campaigns in Nigeria, Democratic Republic of the Congo (DRC), and Ethiopia were likely sources of most cVDPV2 emergences in 2016–2019, with campaigns from a group of 17 the most likely sources of all 46 emergences. From the probabilistic campaign attributions we see a higher emergence risk when the level of OPV-induced immunity in the campaign location is lower, and while larger campaigns carry a greater risk, their per-person risk is smaller. These findings imply cVDPV2 emergence risk per dose to be greatest following the first campaign use of Sabin OPV type 2 in areas without recent use of serotype 2-containing vaccine and when the campaign is small in scale. This suggests that early response to emergences in areas with low immunity should be prioritized to receive nOPV2, a more genetically stable oral polio vaccine given emergency use authorization by WHO in November 2020. Intrinsically, such initial responses are those carried out in the context of lowest immunity, and with the highest risk. If the supply of nOPV is constrained, subsequent campaigns could use Sabin OPV2 in an environment now associated with a lower risk of VDPV2 emergence because of the increase in serotype 2 immunity.

Similarly, it would be advisable to prioritize nOPV2 use for campaigns in locations highly connected to places with low serotype 2 immunity from OPV. It has been observed that in specific locations with a lack of long-range transportation, viral lineages are restricted to a limited geographical region [9]. Conversely, the emergence groups detected in Angola are first detected unusually far from their likely causal campaigns. Of possible relevance here is the 2017 and 2018 conflict in the Kasai region of DRC, leading to large-scale displacement of people to Angola, particularly to Lunda Norte [21], where 3 out of the 5 emergences were first detected.

Outbreak responses should be carried out as soon after detection as possible, in order that the outbreak may be small and stopped by a smaller campaign, rather than necessitating a large or multiple-country campaign. Likewise, the benefit of improved postcampaign surveillance is also implied. If a new outbreak is detected before much spatial spread has occurred, the consequent response may be conducted in a geographical area overlapping the previous campaign area, with a higher level of immunity. Detections of serotype 2 Sabin in nonpolio AFP cases, or environmental sampling in connected places, especially those with low immunity, may serve as early warning signs that an outbreak may be probable.

Our analysis has several limitations, primarily related to the scarcity of the data: risk factor estimates are based on only 46 causal campaigns, the identities of which are highly uncertain. Larger campaigns appear riskier, but there are not enough data to infer a more detailed relationship between campaign size and risk at different immunity levels, as predicted in earlier modeling [4]. As more data become available, it may be possible to include such considerations under the same framework. We are also limited in our ability to identify likely causal campaigns by the lack of temporal resolution of inferred emergence dates based on the molecular clock of the VP1 region, despite its rapid evolution, and the relatively quick succession of campaigns in some regions. This could be addressed in part though whole-genome sequencing, which might improve resolution of the date of cVDPV2 origins by expanding the length of region sequenced from approximately 1 to 7 kilobases. Construction of a full phylogenetic tree and inference of the date of administration of the originating dose for each emergence group may also help by combining data from all detections. We focused only on the first 3 detections because of the nonindependent information provided by each detection as a result of their shared evolutionary history—something that is explicitly captured during phylogenetic reconstruction. There are also limitations in the available risk factor data: esimated of immunity in children younger than five were modelled based on the reported number of doses received by children with nonpolio AFP [14] in the 6-month period in which the campaign fell. These discrete time estimates may lead to over- or underestimation of immunity at the time of potential causal campaigns, depending on their exact timing within that 6-month period. Additionally, these immunity estimates may be biased by inaccurate recall of dose history.

No relationship could be found via our modeling approach between campaign quality data (in the form of LQAS) and risk of emergence. The result is likely due to the lack of data: LQAS data were only available for a quarter of the province-campaigns. Additionally, LQAS may not accurately capture coverage, for example, if there are problems of access in certain locations [22]. This lack of association is not indicative that coverage is irrelevant. From the association of risk with immunity we infer the opposite: if low-quality campaigns are carried out repeatedly in a province, consequent low immunity and repeated exposure of not-immune individuals to vaccinated individuals will lead to increased risk. Likewise, even where we do not have many sequential campaigns, having large numbers of susceptible individuals exposed to vaccinated individuals after a low-quality campaign would likely be a driver of outbreaks, given our findings on campaign size and immunity on emergence risk. In this case, high coverage of campaigns would be important. Despite association of population immunity with cVDPV2 emergence risk, it is likely to explain only a small amount of the variance in risk between campaigns.

The observed distribution of emergences per campaign is highly skewed and it remains unclear why some campaigns in Nigeria, DRC, and Ethiopia caused the majority of new VDPV2 emergences in 2016–2019 in Africa. Other risk factors may be important, such as prevalence of nonpolio enteroviruses acting as recombination partners with OPV, allowing rapid reversion to wild-type neurovirulence and transmissibility [23, 24]. Low immunogenicity of oral vaccines in low-income settings may also contribute, facilitating postcampaign Sabin OPV spread [25, 26]. Nonetheless, our results support the prioritization of nOPV2 for outbreak responses in places of low existing immunity in children younger than five years to have the greatest chance of breaking the response-emergence cycle of cVDPV2 outbreaks.

## Supplementary Data

Supplementary materials are available at *The Journal of Infectious Diseases* online. Consisting of data provided by the authors to benefit the reader, the posted materials are not copyedited and are the sole responsibility of the authors, so questions or comments should be addressed to the corresponding author.

## Supplementary Material

jiad004_Supplementary_Data

## Notes

***Financial support*.** This work was supported by the World Health Organization Polio Research Committee; the Bill and Melinda Gates Foundation (grant number INV-031605); the Medical Research Council (MRC) Centre for Global Infectious Disease Analysis (grant number MR/R015600/1) funded by the UK MRC and the UK Foreign, Commonwealth, and Development Office (FCDO), under the MRC/FCDO concordat agreement and the EDCTP2 Program supported by the European Union; and Community Jameel.
