# Supplementary material for: The Origins and Risk Factors for Serotype-2 Vaccine-Derived Poliovirus Emergences in Africa During 2016–2019
Source: J Infect Dis. 2023 Jan 11;228(1):80–8. doi: 10.1093/infdis/jiad004 (PMC10304761; doi:10.1093/infdis/jiad004)
Supplement: jiad004_Supplementary_Data [file jiad004_supplementary_data.pdf]

Origins and risk factors for serotype-2 vaccine-derived poliovirus (VDPV2) outbreaks in Africa 2016-2019: technical details.

Elizabeth J. Gray, Laura V. Cooper, Ananda S. Bandyopadhyay, Isobel M. Blake, and Nicholas C. Grassly

1 Model Description

Notation

Say we have n outbreaks, outbreak i of which consists of n_i cases, found in locations $l_1^i, \ldots, l_{n_i}^i$ at times $t_1^i, \ldots, t_{n_i}^i$, having $u_1^i, \ldots, u_{n_i}^i$ nucleotide changes from Sabin. Our aim for each of these outbreaks is to construct a probability distribution over the space of vaccination campaigns which will give the probability that each campaign was that from which the outbreak was seeded. We define the random variable $\boldsymbol{C} = (C_1, \ldots, C_n)$ to be the set of seeding campaigns. Say there are N such campaigns: $\boldsymbol{S} = (S_1, \ldots, S_N)$ with campaign S_j being carried out in province L_j at time T_j. The elements of $\boldsymbol{C}$ may take as values any of the elements of $\boldsymbol{S}$. Locations are considered at the province level (i.e. a location refers not to a point in space: the population weighted centroid of that province, rather than a whole province). We denote the proportion of the whole-SIA, of which it is a constituent part, covered by province-SIA j by w_j. This ensures the results are not sensitive to arbitrary province divisions within SIAs.

We begin by describing each component of the hierarchical model, which fit together as displayed in Figure 1, as will be described later.

Probability of seeding based on risk factors

Given covariates $\boldsymbol{x}^j = (x_{u5,j}, x_{dp,j}, x_{LQAS,j}, \log(x_{size,j}))$ associated with campaign S_j, and unknown parameters $\boldsymbol{\theta} = (\theta_{u5,j}, \theta_{dp,j}, \theta_{LQAS,j}, \theta_{size,j})$, the probability that province campaign j was the causing-SIA of outbreak i is assumed to be

$$\mathrm{P}(C_i = S_j | \boldsymbol{\theta}, \boldsymbol{X}) = \frac{w_j \exp\left(\boldsymbol{\theta}^T \boldsymbol{x}^j\right)}{\sum_{q=1}^N w_q \exp\left(\boldsymbol{\theta}^T \boldsymbol{x}^q\right)}, \tag{1}$$

and thus we denote the probability distribution of the whole vector $\boldsymbol{C}$ as

$$\boldsymbol{P}(\boldsymbol{C}|\boldsymbol{\theta}, \boldsymbol{X}) = \frac{\exp(\sum_{i=1}^n \log(w_{i^*}) + \boldsymbol{\theta}^T \boldsymbol{x}^{i^*})}{\left(\sum_{q=1}^N w_q \exp\left(\boldsymbol{\theta}^T \boldsymbol{x}^q\right)\right)^n}, \tag{2}$$

where i^* is the value of j s.t $C_i = S_j$.

The use of the product of categorical distributions in Equation 2 implies that we have conditioned on the number of outbreaks that have occurred. This is necessitated by the fact that in order for us to make use of the relationship between the difference in time between the camapaign and detection, and the number of nucleotide changes found, we must condition on the set of numbers of nucleotide changes: i.e. conditional on the fact that a specific set of cases or detection with their associated set of numbers of nucleotide changes will definitely be found, we find the likelihood of the time and location at which it is found, given each possible seeding campaign. Note that Equation 2 implies that given the covariates and parameters, the emergence of each distinct group is independent of all others, an assumption which would be violated by groups of

emergence groups sharing common ancestors (i.e. these emergences should, in terms of campaign attribution, be considered just one emergence). This may lead to poor estimation in the parameters $\boldsymbol{\theta}$. Future investigations involving genetic data may enable dispensing with this assumption, in the simplest case by treating emergence groups with such a known common ancestor as a single emergence.

Spatial spread

An adjusted version of the radiation model for population movement of [1] is used to account for spatial spread. The radiation model gives an estimate of the population 'flux' between any two locations based on their own populations, and the populations surrounding them. Denoting the population of province i by m_i, and the population enclosed in the circle centred at i, the circumference of which touches k, not including the populations of i and k, by s_{ik}, we define, for $i \neq k$

$$q^*_{ik} = \frac{m_i m_k}{(m_i + s_{ik})(m_i + m_k + s_{ik})}, \tag{3}$$

$$q_{ik} = \frac{q^*_{ik}}{\sum_{j \neq k} q^*_{jk}}. \tag{4}$$

An unknown 'province retention' parameter β is introduced to represent the slowness of spatial spread, which may be interpreted as the probability that any one transmission is between two people from the same province. Thus the one-generation transition matrix R may be defined, with elements

$$R_{ik} = \begin{cases} (1-\beta)q_{ik} & \text{if } i \neq k \\ \beta & \text{otherwise.} \end{cases} \tag{5}$$

Given a number of generations g^i_k between seeding and detection for case k of outbreak i, and a seeding campaign $C_i = S_j$, the likelihood of the location l^i_k is given by

$$P(l^i_k | C_i = S_j, g^i_k, \beta) = \left(R^{g^i_k}\right)_{L_j, l^i_k}, \tag{6}$$

where the matrix exponentiation takes accounts for all possible routes the chain of infection could have taken between the seeding location and the detection location, over the g^i_k generations.

The computation of many matrix powers of R for every new value of β, within an MCMC context is very computationally expensive, and carries the greatest computational burden of the model fitting process. It is useful to note that R is of the form $R = \beta I + (1-\beta)Q$, (where Q is the normalised radiation model matrix with zeros on the diagonal). This means, by the commutativity of these matrices and the binomial theorem, we have

$$R^n = \sum_{k=0}^{n} \binom{n}{k} \beta^k (1-\beta)^{n-k} Q^{n-k}, \tag{7}$$

the computation of which is considerably faster than other methods of matrix exponentiation, as the powers of the matrix Q may be pre-calculated. Further time savings may be made here where parallelisation is possible, as each power of R may be calculated independently.

The number of generations g^i_k, through which case k of outbreak i has been transmitted, is not known, but given its probability distribution, conditioned on the time between seeding and detection, we can marginalise as follows:

$$P(l^i_k | C_i = S_j, t^i_k, \beta, \boldsymbol{\zeta}_\gamma) = \sum_{g^i_k = 0}^{\infty} P(l^i_k | C_i = S_j, g^i_k, \beta) P(g^i_k | t^i_k, \boldsymbol{\zeta}_\gamma, C_i = S_j). \tag{8}$$

N.B. in practice the sum to infinity cannot be computed, but instead may be restricted to terms beyond which the remaining probability mass is very close to zero. We choose via simulation a maximum number of generations m such that the observation with the single largest number of nucleotide changes has a less than one percent chance of having gone through more than m transmission generations. As the matrix power step is the most computationally expensive, there is a computation time vs. accuracy trade off in increasing the value of m, however, we note that accuracy gains from making m arbitrarily large are likely to be absorbed by MCMC error.

Time and number of generations

The time for one generation (the time between two successive infections) is assumed to follow a gamma distribution with shape and rate parameters $\boldsymbol{\zeta}_\gamma = (\alpha_\gamma, \beta_\gamma)$. These are taken to be (on the week scale) 1.43 and 1 respectively. This corresponds to an expected generation time of ten days, (1.43 weeks), as in [2].

We assume that the time for one generation follows a gamma distribution with shape and rate parameters $\boldsymbol{\zeta}_\gamma = (\alpha_\gamma, \beta_\gamma)$. We wish, given a length of time t, to derive a probability distribution for the number of generations through which the virus has gone during that time. Say we denote the length of the ith generation by v_i. We have

$$v_i \sim \text{gamma}(\alpha_\gamma, \beta_\gamma), \tag{9}$$

and, by the properties of the gamma distribution, and the independence of the generation times

$$v_1 + \cdots + v_n \sim \text{gamma}(n\alpha_\gamma, \beta_\gamma). \tag{10}$$

The probability that after a time interval of t there have been exactly n complete generations may be expressed as

$$\text{P}\left(v_1 + \dots v_n < t < v_1 + \dots v_{n+1}\right) = \text{P}\left(t > v_1 + \dots v_n\right) - \text{P}\left(t > v_1 + \dots v_{n+1}\right). \tag{11}$$

As $\text{P}\left(t > v_1 + \dots v_n\right)$ is simply one minus the cumulative distribution function of a gamma distribution with parameters $n\alpha$ and β, we have

$$P(n \text{ generations}|t, \boldsymbol{\zeta}_\gamma) = \frac{\gamma(\alpha_\gamma(n+1), \beta_\gamma t)}{\Gamma(\alpha_\gamma(n+1))} - \frac{\gamma(\alpha_\gamma n, \beta_\gamma t)}{\Gamma(\alpha_\gamma n)}, \tag{12}$$

where Γ and γ denote the complete and incomplete gamma functions, respectively. This means that we have

$$p(g_k^i = n|t_k^i, \boldsymbol{\zeta}_\gamma, C_i = S_j) = \frac{\gamma(\alpha_\gamma(n+1), \beta_\gamma(t_k^i - T_j))}{\Gamma(\alpha_\gamma(n+1))} - \frac{\gamma(\alpha_\gamma n, \beta_\gamma(t_k^i - T_j))}{\Gamma(\alpha_\gamma n)}. \tag{13}$$

The time between nucleotide changes is assumed to follow an exponential distribution with rate λ as in [3], and so the time t since seeding follows an Erlang distribution with rate $\lambda = 0.1970$ (on the week scale) and shape parameter u the number of nucleotide changes. This means that the likelihood of the time associated with case k of outbreak i, assuming that it was seeded by province-SIA j is given by

$$p_t(t_k^i|u_k^i, C_i = S_j) = \begin{cases} \frac{\lambda^{u_k^i}(t_k^i - T_j)^{u_k^i - 1}\exp(-\lambda(t_k^i - T_j))}{(u_k^i - 1)!} & \text{if } t_k^i - T_j > 0 \\ 0 & \text{otherwise.} \end{cases} \tag{14}$$

Overall Model

The above relationships between variables are visualised in Figure 1. Putting all model components together, and marginal-

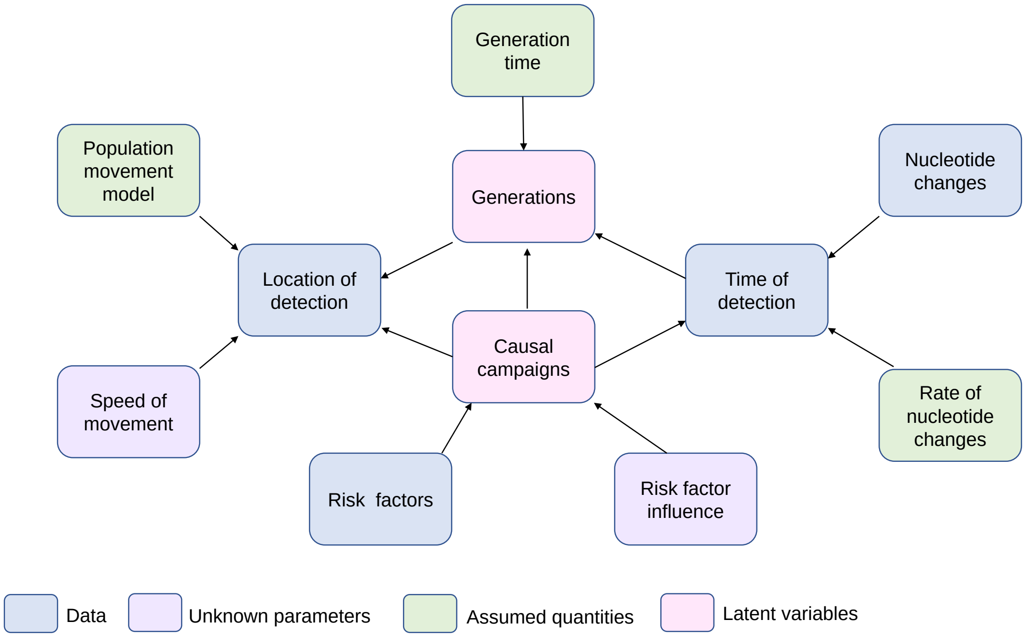

Figure 1: Directed acyclic graph describing relationships between variables, data and assumed known quantities. N.B. The direction of the arrow between 'time of detection' and 'nucleotide changes' (indicating that, given the number of nucleotide changes and rate, time since detection follows and Erlang distribution) could be switched, and the arrows from 'rate of nucleotide changes' and 'causal campaigns' to 'time of detection' switched to point to 'nucleotide changes', indicating instead that the number of nucleotide changes given the time of detection and rate of change follows a Poisson distribution: both formulations are equivalent. For the purposes of modelling, one of either time or nucleotide changes must be considered as fixed, and the other a dependent variable.

ising over the number of transmission generations (as in Equation 8) we obtain the full joint distribution:

$$P(\boldsymbol{C}, \boldsymbol{l}, \boldsymbol{t}, \boldsymbol{\theta}, \beta | \boldsymbol{X}, \boldsymbol{\zeta}_\gamma, \boldsymbol{u}, \lambda, \alpha_\beta, \beta_\beta, \boldsymbol{\zeta}_\gamma, \boldsymbol{\mu}_\theta, \boldsymbol{\Sigma}_\theta) = P(\beta|\alpha_\beta, \beta_\beta) P(\boldsymbol{\theta}|\boldsymbol{\mu}_\theta, \boldsymbol{\Sigma}_\theta) P(\boldsymbol{C}|\boldsymbol{\theta}, \boldsymbol{X}) \\ P(\boldsymbol{t}|\boldsymbol{C}, \boldsymbol{u}, \lambda) P(\boldsymbol{l}|\beta, \boldsymbol{t}, \boldsymbol{\zeta}_\gamma, \boldsymbol{C}), \quad (15)$$

where

$$P(\boldsymbol{t}|\boldsymbol{C}, \boldsymbol{u}, \lambda) = \prod_{i=1}^{n} \prod_{k=1}^{n_i} p_t(t_i^k | u_k^i, C_i, \lambda), \quad (16)$$

$$P(\boldsymbol{l}|\boldsymbol{t}, \beta, \boldsymbol{C}, \boldsymbol{\zeta}_\gamma) = \prod_{i=1}^{n} \prod_{k=1}^{n_i} p_l(l_k^i | t_k^i, C_i, \beta, \boldsymbol{\alpha}). \quad (17)$$

The conditional posterior distributions for the causal campaigns $\boldsymbol{C}$, risk factor parameters $\boldsymbol{\theta}$ and movement parameter β are proportional to this joint distribution. In the following section we will describe a Markov Chain Monte Carlo (MCMC) procedure by which iterative draws from these conditional posteriors may be made, in order to find posterior distributions for $\boldsymbol{C}$, $\boldsymbol{\theta}$ and β.

2 Model fitting

This model was fit to the set of detections and province-SIAs with five MCMC chains of length 11000 with the first 1000 discarded. For every sample taken of the movement parameter β and the set of causal campaigns, we take the 80th sample of $\boldsymbol{\theta}$ to improve chain mixing. A Beta prior is assumed on β with hyperparameters $(20, 2)$, concentrating most of the probability mass between 0.9 and 1, as we expect nearly all transmissions to be among people from the same province. We

assume standard Gaussian priors on the elements of $\boldsymbol{\theta}$, with the exception of θ_{size}, the prior mean of which we specify as 1 (i.e. a linear relationship between risk and campaign size). The resulting output is a sample of size 50000 from the posterior distributions of the causal campaigns $C_1, \ldots, C_n$, and the parameters β and $\boldsymbol{\theta}$.

2.1 Model fitting procedure

Here we outline the Markov chain Monte Carlo (MCMC) routine implemented to fit the model. Where parallel computing is possible, this may be implemented across several simultaneous chains to improve efficiency. For n^* the number of cases, n the number of outbreaks, and N the number of province SIAs, we proceed as follows:

1. Intialisation with starting values β^0, $\boldsymbol{\theta}^0$:

   (a) Generate transition matrix $R(\beta^0)$.

   (b) Find powers $R^0(\beta^0), \ldots, R^m(\beta^0)$, where m is the largest possible number of generations any of the cases could reasonably have been through (within some small level of tolerance).

   (c) For each case-province-SIA pair, and corresponding time gap $(t_i^k - T_j)$, find the probability distribution of the number of generations $P(g_{ij}^k = g|(t_i^k - T_j), \boldsymbol{\zeta}_\gamma)$, $g = 0, \ldots, m$. Store these in a list of m $n^* \times N$ matrices $G_1, \ldots, G_m$. These will stay constant throughout the MCMC routine.

   (d) For each case-province-SIA pair, marginalise over the generations to find the likelihood of each detection location, given the province-SIA:

   $$\sum_{g=0}^{m} R^g(\beta^0)_{L_j, l_i^k} P(g_{ij}^k = g|(t_i^k - T_j), \boldsymbol{\zeta}_\gamma).$$

   Store these values in an $n^* \times N$ matrix L^*. Shrink L^* down to an $n \times N$ matrix by taking products such that

   $$L_{ij} = \prod_{k \in V_j} L_{ik}^*$$

   where $V_j$is the set of all cases relating to outbreak j

   (e) For each case-province-SIA pair, find the likelihood of the time gap $(t_i^k - T_j)$, assuming an Erlang distribution, with the rate the number of nucleotide changes u_i^k. Store these in an $n^* \times N$ matrix T^*. Shrink T^* down to a matrix of size T (in the same way as $L^* \to L$) by taking products of the elements pertaining to cases from the same outbreak. This matrix T stays constant throughout the MCMC routine.

   (f) Given $\boldsymbol{\theta}^0$ find a length N vector $B(\boldsymbol{\theta}^0)$ such that

   $$B_k(\boldsymbol{\theta}) = \frac{w_k \exp(\boldsymbol{\theta}^T \boldsymbol{x}^k)}{\sum_{j=1}^{N} w_k \exp(\boldsymbol{\theta}^T \boldsymbol{x}^k)}$$

   (g) The posterior probability given the initial values, that outbreak i has come from campaign j is then proportional to the product

   $$B_j(\boldsymbol{\theta}^0) \times T_{ij} \times L(\beta^0)_{ij}.$$

   Use these values to sample a starting value of the vector $\boldsymbol{C}^0$.

2. For $a = 1, \ldots$

   (a) Sample β^a:

i. Propose a new value β^* from a distribution $Q(\beta|\beta^{a-1})$.

ii. Construct $R(\beta^*)$ and $L(\beta^*)$.

iii. Set $\beta^a = \beta^*$ with probability

$$\min\left\{1, \frac{\prod_{i=1}^{n} L(\beta^*)_{i,C_i^{a-1}} P(\beta^*|\alpha_\beta, \beta_\beta) Q(\beta^{a-1}|\beta^*)}{\prod_{i=1}^{n} L(\beta^{a-1})_{i,C_i^{a-1}} P(\beta^{a-1}|\alpha_\beta, \beta_\beta) Q(\beta^*|\beta^{a-1})}\right\}$$

else set $\beta^a = \beta^{a-1}$.

(b) Sample $\boldsymbol{C}^a$ as in step 1(g) using $B(\boldsymbol{\theta}^{a-1})$, T, $L(\beta^a)$.

(c) Sample $\boldsymbol{\theta}^a$:

i. Introduce auxiliary variable $\boldsymbol{\theta}^x = \boldsymbol{\theta}^{a-1}$.

ii. Construct $B(\boldsymbol{\theta}^x)$.

iii. For each element $y = 1 \ldots$ of $\boldsymbol{\theta}$

A. Propose θ_y^* from a Gaussian random walk centred at θ_y^x.

B. Construct $\boldsymbol{\theta}^*$ s.t. $\theta_y^* = \theta_y^*$ and $\theta_i^* = \theta_i^x$ for $i \neq y$, and vector $B(\boldsymbol{\theta^*})$.

C. Set $\boldsymbol{\theta^x} = \boldsymbol{\theta^*}$, and $B(\boldsymbol{\theta^x}) = B(\boldsymbol{\theta}^*)$ with probability

$$\min\left\{1, \frac{\prod_{i=1}^{n} B(\boldsymbol{\theta^*})_{C_i^a}}{\prod_{i=1}^{n} B(\boldsymbol{\theta^x})_{C_i^a}} \frac{P(\theta_y^*|\mu_{\theta,y}, \sigma_{\theta,y}^2)}{P(\theta_y^x|\mu_{\theta,y}, \sigma_{\theta,y}^2)}\right\}$$

iv. Having iterated through 2(c)(iii) one or more times, set $\boldsymbol{\theta^a} = \boldsymbol{\theta^x}$

(d) Repeat from step 2(a).

3 Expected distances and times between emergence and detection

In order to estimate the expected time delay and distance travelled between an emergence-causing campaign and its first detection, we find the probability-weighted mean of the distances between the population weighted centroid of each province campaign and the centre of the district at which the first detection was found (the locations are not available in any finer detail due to personal identifiability concerns). A similar calculation is made for the times. For example, if an emergence were first detected a distance of d_a and d_b from the province weighted centroids of campaign provinces a and b, at times t_a and t_b after they had been carried out, and we estimated that the probabilities of having been the causal campaign were 60% and 40% for a and b respectively, then the expected distance would be $0.6m_a + 0.4m_b$ and time $0.6t_a + 0.4t_b$.

4 Risk factors and emergence risks

To visualise the relationships between emergence causation probability and these risk factors we can find the per-person risk of seeding each emergence for every province-SIA. This ad hoc per person measure of risk enables us to make comparisons possible across campaigns of different sizes by dividing by the total population of each campaign area. We then sum these over all 46 emergences to find a per-person aggregate risk, which we use as an indication of campaign risk, adjusted for size. We subset the province-SIAs according to their risk factor values and take the mean aggregate risk for each group. These are shown in Figure 2. Similarly we find the fitted per-person risk based only on the prior distribution, with the posterior mean estimates for θ_{u5} and θ_{size}. By per-person, we mean the total population of that province, rather than the number of doses given.

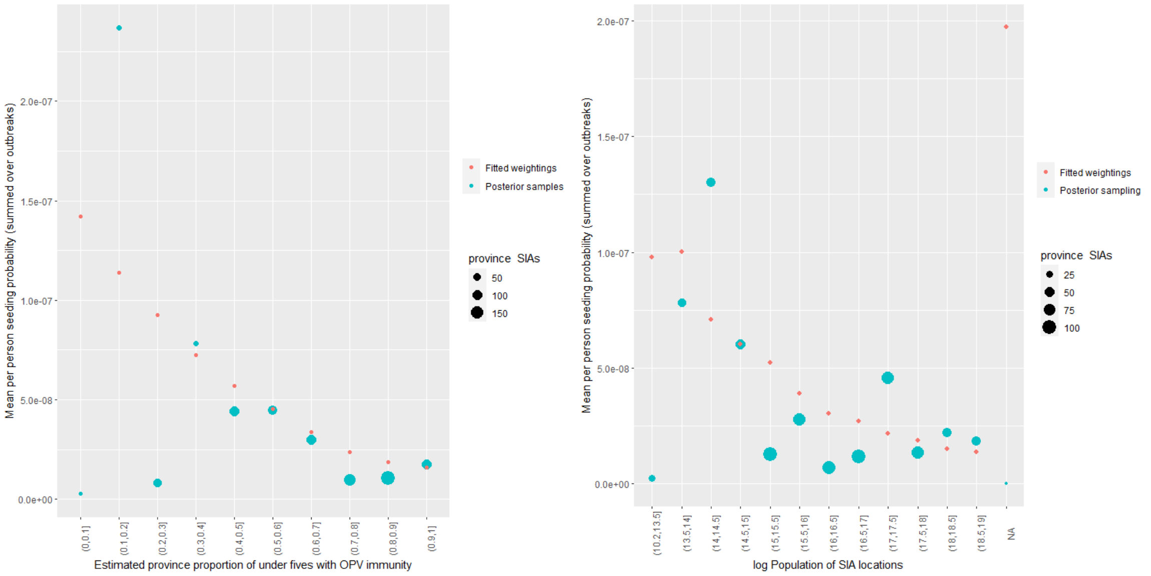

Figure 2: Mean value of per-person emergence causation probabilities (summed over all emergences) for all province-SIAs, subsetted by log size and immunity, calculated from the posterior samples of campaigns $\boldsymbol{C}$. Also the mean prior per person probabilities, again summed over all emergences for each of these province-SIAs.

4.1 Estimated absolute risk of emergence

While the parameters θ_{u5} and θ_{size} inform the relative risks of a new outbreak being caused, we can use the number of outbreaks (46) to find an estimate of the expected number of outbreaks caused by (possibly new) campaign, given its size $x_{size,i}$ and immunity $x_{u5,i}$, and the set C of campaigns. We first find the per-person risk pp_i:

$$pp_i = \hat{a} \exp\left((\theta_{size} - 1)\log(x_{size,i}) + \theta_{u5} x_{u5,i}\right) \tag{18}$$

where

$$\hat{a} = \frac{46}{\sum_{j \in C} \exp\left(\theta_{size} \log(x_{size,j}) + \theta_{u5} x_{u5,j}\right)}. \tag{19}$$

Thus expected number of new outbreaks caused by this campaign would be given by $pp_i x_{size,i}$. These can be used to construct estimates that a campaign will produce at least one outbreak, assuming that outbreaks occur as a Poisson process, with rate parameter $pp_i x_{size,i}$.

References

[1] Filippo Simini, Marta C González, Amos Maritan, and Albert-László Barabási. A universal model for mobility and migration patterns. *Nature*, 484(7392):96–100, 2012.

[2] Neal Nathanson and Olen M Kew. From emergence to eradication: the epidemiology of Poliomyelitis deconstructed. *American journal of epidemiology*, 172(11):1213–1229, 2010.

[3] GR Macklin, KM O'Reilly, NC Grassly, WJ Edmunds, O Mach, R Santhana Gopala Krishnan, A Voorman, JF Vertefeuille, J Abdelwahab, N Gumede, et al. Evolving epidemiology of Poliovirus serotype 2 following withdrawal of the serotype 2 oral Poliovirus vaccine. *Science*, 368(6489):401–405, 2020.
